# Apatinib and temozolomide in children with recurrent ependymoma: A case report

**DOI:** 10.1097/MD.0000000000030529

**Published:** 2022-09-16

**Authors:** Shuangshuang Zhao, Zhipeng Shen, Juan Li, Lei Shi, Ni Zhang

**Affiliations:** a Department of Radiation Oncology, Hangzhou Cancer Hospital, Hangzhou, Zhejiang, P.R. China; b Department of Neurosurgery, The Chilidren’s Hospital, Zhejiang University School of Medicine, Hangzhou, Zhejiang, PR China.

**Keywords:** apatinib, recurrent pediatric ependymomas, targeted therapy, temozolomide, vascular endothelial growth factor receptor

## Abstract

Outcomes remain poor in children with recurrent ependyomams (rEPNs), despite advances in surgery and radiotherapy. Systemic therapeutic options are limited, given the low response to chemotherapy and targeted drugs. There is an urgent need for efective pharmacotherapy. Apatinib is a multitarget tyrosine kinase inhibitor, which has been reported to exhibit broad antitumor profiles. However, its effects on rEPNs have not been reported thus far.

**Patient concerns:**

We present a 5-year-old recurrent ependyomam patient benefting from apatinib and temozolomide. The patient was diagnosed with ependyomam in January 2016 and treated with surgery and radiotherapy. After surgery, the patient walked with an mild unsteady gait. He was diagnosed with recurrence in November 2018 following which he was treated with reoperation, reirradiation and chemotherapy (etopside, cisplatin, and temozolomide [TMZ]). The patients increased gait instability in April 2019.

**Diagnoses:**

Magnetic resonance imaging (MRI) showed progression of the disease. The lession at the left edge of the fourth ventricle and cerebellar peduncles was significantly increased.

**Interventions:**

The patient was administer TMZ (200 mg/m^2^/d, d1-5, 28 days as a cycle) + apatinib (250 mg, every other day). Twelve cycle of TMZ and apatinib were given.

**Outcomes:**

The tumor significantly shrank during the patient received TMZ and apatinib. After 9 months of medication, MRI revealed a nearly complete response However, the tumor progressed on May 5, 2020. From the beginning of the application of TMZ and apatinib, the progression-free survival was 1 year and the survival time was 19 months. Grade 1 leukocytopenia was observed without other adverse effects.

**Conclusion:**

Apatinib and temozolomide treatment with mild side effects may be a new option for children with recurrent ependyomams.

## 1. Introduction

Ependymomas (EPNs) arise from the lining of the ventricles of the central nervous system (CNS). These tumors account for 5.2% of all primary CNS tumors in pediatric patients^[1]^ (0 to 19 years of age). In pediatric intracranial EPNs of WHO grade II or III, surgery followed by local radiotherapy remains the best therapy for newly diagnosed disease. However, cases of recurrent ependymomas (rEPNs) continue to have poor outcomes and standard salvage options have not been identified. The European Association of Neuro-Oncology^[2]^ (EANO) has recommended salvage therapies for rEPNs, including reoperation, reirradiation, and chemotherapy. For pediatric rEPNs, phase II studies^[3]^ have reported a response rate to single agents of 11%, with 4.6% complete response (CR); the response rate of combinations and high-dose chemotherapy was 26% with 12% CR. Additional therapeutic options are urgently needed for pediatric rEPNs.

Similar to other gliomas, EPNs overexpress vascular endothelial growth factor (VEGF) and vascular endothelial growth factor receptors (VEGFRs); this overexpression has been correlated with poor survival.^[4,5]^ VEGF/VEGFR signaling plays an important role in tumor angiogenesis, which is an essential step in tumor growth and metastasis. VEGFR-2 plays a central role in VEGF-induced angiogenic signaling.^[6]^ Apatinib (Hengrui Pharmaceutical Co. Ltd., Jiangsu, China), an oral small molecular tyrosine kinase inhibitor (TKI) that selectively targets the intracellular domain of VEGFR-2,^[7]^ was first approved for third-line treatment of advanced gastric cancer patients.^[8]^ However, apatinib has been demonstrated to have broad antitumor profiles, which include effects on advanced nonsquamous nonsmall-cell lung cancer,^[9]^ breast cancer,^[10]^ and hepatocellular carcinoma.^[11]^ Recently, a Phase III trial showed that apatinib combined with temozolomide (TMZ) resulted in a significant improvement in progression-free survival (PFS) and overall survival (OS) for recurrent glioblastoma.^[12]^ Until now, there have been no reports of the treatment of rEPN with apatinib. The efficacy and security of children with refractory rEPN after taking apatinib and TMZ was reported in this study.

## 2. Materials and methods

### 2.1. Ethics statement

The Ethics Committee of the Hangzhou Cancer Hospital approved the study.

### 2.2. Case report

**Figure 1.**
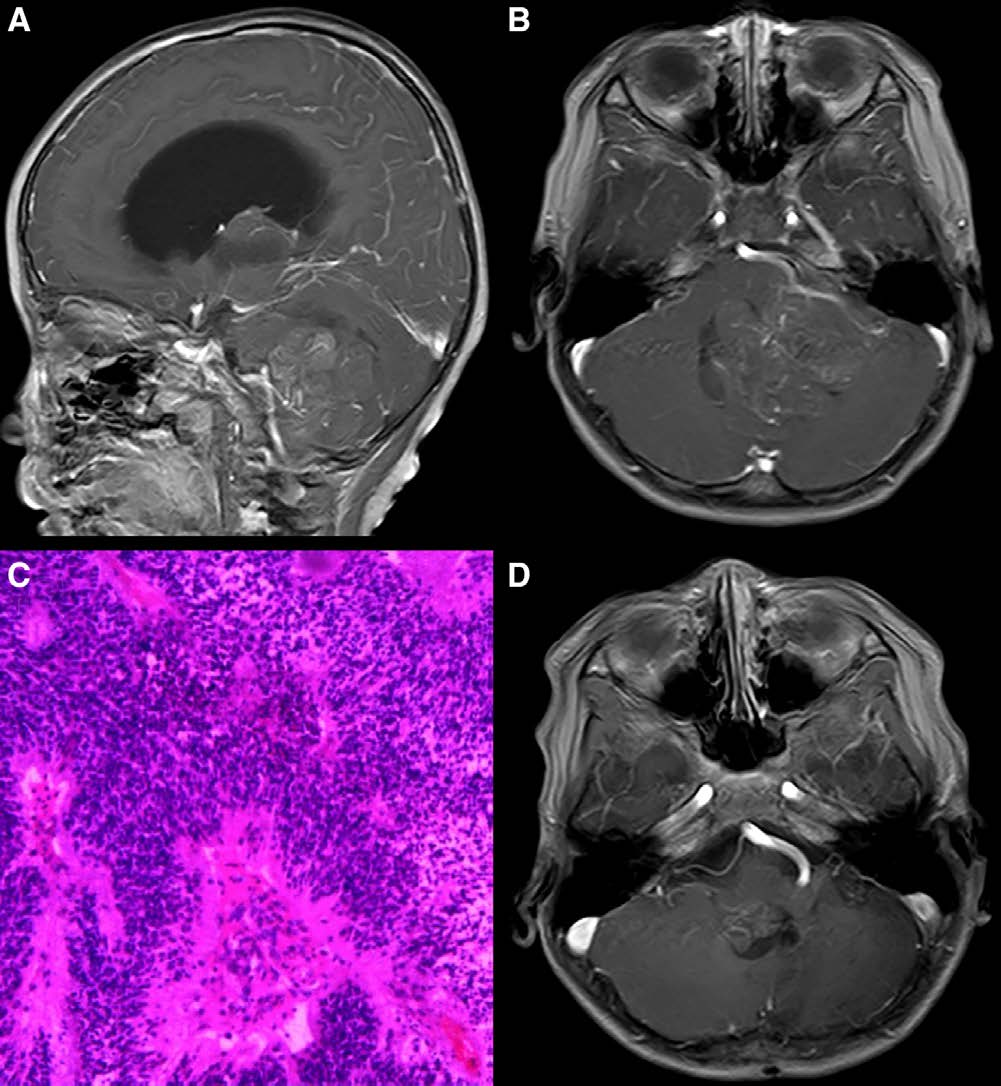
Pre- and postoperative MRI and pathological diagnosis. Notes: (Figure A, sagittal image and figure B, coronal image) Preoperative T1 contrast-enhanced axial images showed a space-occupying lesion with heterogeneous intensity and nonhomogeneous enhancement in the left cerebellum and fourth ventricle. (C) Postoperative histopathological section (HE staining, magnification x100) showed features of ependymoma (WHO grade II) with increased cellularity, nuclear atypia, and mitotic activity, arranged in perivascular pseudorosettes. (D) At 34 months after the first operation, T1 contrast-enhanced axial image showed a mass with nonhomogeneous enhancement at the right margin of the fourth ventricle. Abbreviations: MRI = magnetic resonance imaging.

A 26-month-old patient presented to a chilidren’s hospital for more than a half month of gait ataxia without dizziness and vomiting. Magnetic resonance imaging (MRI) revealed a large space-occupying lesion in the left cerebellum and fourth ventricle with obstructive hydrocephalus (Fig. 1A and B). The patient underwent total resection on January 22, 2016 and was pathologically diagnosed with ependymoma (WHO grade II) (Fig. 1C). Immunohistochemistry examination revealed the following: GFAP (+), VIM (+), EMA (+), INI-1 (+), CK (-), CD99 (-), and Ki67 (overall + 5–10%, focal + 20 to 30%). After surgery, the patient walked with an mild unsteady gait. After recovering from surgery, the patient was referred to our hospital to receive intensity-modulated radiation therapy (IMRT) with 50 Gy in 25 fractions for tumor bed. An MRI scan showed a mass with nonhomogeneous enhancement at the right fourth ventricle in November 2018 (Fig. 1D). Considering the new mass was at the right edge of the operative cavity, the patient was clinically diagnosed with rEPN. A second operation to resect the new lesion was performed on November 26, 2018, and postoperative histopathology remained the same. Then, the patient was treated with IMRT with 45 Gy in 25 fractions for tumor bed.

**Figure 2.**
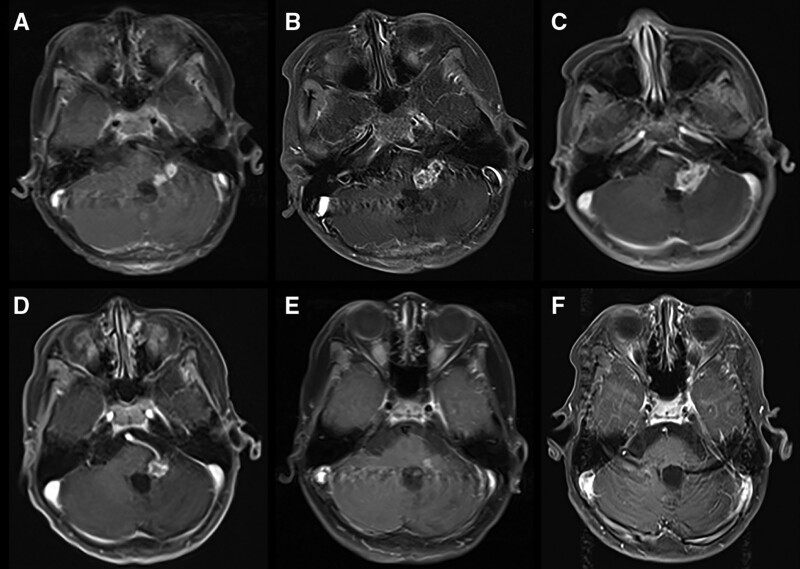
Chronological changes on magnetic resonance imaging (MRI) show second tumor recurrence and response. Notes: Coronal MRI of T1 contrast-enhanced axial images were taken at the following times: (A) diagnosis of relapse of the second tumor, (B) after 2 cycles of EP, (C) after 1 cycle of TMZ + DDP, (D) after 5 months of TMZ + apatinib, (E) after 7 months of TMZ + apatinib, and (F) after 9 months of TMZ + apatinib. Abbreviations: EP = etopside + cisplatin, TMZ + DDP = TMZ + cisplatin, MRI = magnetic resonance imaging, EPNs = Ependymomas, CNS = central nervous system, rEPNs = recurrent ependymomas, EANO = European Association of Neuro-Oncology, CR = complete response, VEGF = vascular endothelial growth factor, VEGFRs = vascular endothelial growth factor receptors, TKI = tyrosine kinase inhibitor, TMZ = temozolomide, PFS = progression-free survival, OS = overall survival.

One week after the end of reradiotherapy, MRI showed multiple abnormal enhancements at the left edge of the fourth ventricle and cerebellar peduncles (Fig. 2A), which was considered as the second tumor relapse. After radiotherapy failure, the patient began to receive an EP chemotherapeutic regimen (etopside, 80 mg/m^2^/d + cisplatin, 15 mg/m^2^/d, intravenous administration, d1–5, repeated every 21 days). After 2 cycles of EP, MRI scans indicated a progressive disease and showed an increase in tumor volume (Fig. 2B). Then, TMZ and DDP (TMZ, 100 mg/m^2^/d + cisplatin, 15 mg/m^2^/d, intravenous administration, d1–5, repeated every 28 days) were given to the patient. However, 1 month later, MRI showed a progressive increase in lesion size (Fig. 2C). The patients increased gait instability in April 2019. There were no further standard drugs suitable for this patient. Apatinib, an oral small molecule TKI, shows broad antitumor profiles, including effects against recurrent glioblastoma. His parents signed the informed consent and the patient accepted TMZ (200 mg/m2/d, d1-5, oral administration, 28 days as a cycle) + apatinib (250 mg, every other day, oral administration). After 5 months of treatment, MRI scans indicated a partial response on September 20, 2019 (Fig. 2D). MRI after 7 months of the combined therapy showed that the nodular lesion had shrunk even further (Fig. 2E) and after 9 months of medication, MRI revealed a nearly complete response (Fig. 2F). PFS was 1 year until a new lesion in the right fourth ventricle was found on May 5,2020. After that, he was given the best support treatment. The patient presented with difficulty walking and headache in November 2020. In the end, he died on November 29, 2020. During TMZ + apatinib treatment, the patient experienced grade 1 leukocytopenia, and did not exhibit hand-foot-syndrome, stomatitis, or hypertension. Throughout the course of the disease, the efficacy and tolerability of TMZ + apatinib was sufficient for this patient with rEPN.

## 3. Discussion

EPNs are rare CNS tumors. The annual incidence of EPNs is estimated at 0.43 per 100,000 population in America.^[1]^ EPNs is more common in children than in adults, but the prognosis is worse. Following curative treatment, pediatric rEPNs recur in up to 23 to 66% of patients.^[13]^ The median PFS from date of recurrence is 6.7 months and the median OS is 11.2 months.^[13]^ Until now, there has been no standard therapy at the time of recurrence. The EANO^[2]^ has recommended salvage therapies to treat rEPNs, including reoperation, reirradiation, and chemotherapy. Among pediatric rEPNs, there was a 5-year event free survival of 19% in case of re-gross total resection and 14% in case of incomplete resection.^[14]^ reirradiation can also achieve a durable response.^[15]^ However, the role of chemotherapy in the treatment of pediatric rEPNs remains unclear, which is considered only when surgery and radiotherapy have failed. Phase II studies^[3]^ have reported a low response rate to single agents of 11% with 4.6% CR; combinations and high-dose chemotherapy had a response rate of 26% with 12% CR. The patient described in this study experienced a second relapse after reoperation and reradiotherapy and chemotherapy regimens (etopside, cisplatin, and TMZ) were ineffective.

Several studies have confirmed the antitumor activity of the small molecule TKI apatinib. The antitumor mechanism of apatinib includes many aspects.^[16]^ First, apatinib can suppress angiogenesis and vascular mimicry. Second, apatinib can stimulate apoptosis and prevent proliferation of tumor cells. Third, apatinib can reverse cancer multidrug resistance and sensitize resistant tumor cells to chemotherapy. The most important mechanism is the suppression of angiogenesis, for which apatinib can compete for the adenosine triphosphate binding site of intracellular VEGFR-2 to block downstream signaling and inhibit tumor angiogenesis.^[7]^ In vitro, apatinib can effectively inhibit proliferation, migration, and tube formation of human umbilical vein endothelial cells, and block budding of the rat aortic ring.^[7]^ Moreover, apatinib can also mildly inhibit c-Kit, c-Scr, and Ret, exerting direct antitumor effects.^[7]^ Recently, a Phase III trial showed that apatinib combined with TMZ resulted in a significant improvement of PFS and OS for recurrent glioblastoma.^[12]^ A preclinical study^[17]^ showed that apatinib significantly inhibited cell proliferation, colony formation, invasion, and migration via downregulation of expression of phosphorylated VEGFR2, Akt, and Erk in glioma cells (U251MG and U-87MG). Notably, apatinib plus TMZ resulted in enhanced antitumor effects compared with TMZ alone.^[17]^ This patient exhibited resistance to chemotherapy, while treatment with apatinib and TMZ resulted in obvious tumor regression. These findings indicate a synergistic antitumor effect of apatinib with TMZ.

EPNs are highly vascular tumors; the vascular density of EPNs is similar to that of glioblastoma.^[18]^ EPNs overexpress VEGF and VEGFRs, which has been correlated with poor survival.^[4,5]^ The VEGF-neutralizing antibody bevacizumab has been shown to demonstrate no efficacy in pediatric rEPNs.^[19]^ Other clinical studies have reported a lack of efficacy of bevacizumab in association with irinotecan^[20]^ and lapatinib.^[21]^ Sunitinib, a small multitarget TKI that inhibits VEGFR, PDGRF, and c-Kit, also failed to show activity.^[22]^ The efficacy of targeted drugs in these patients is disappointing. Why dose apatinib with TMZ have such a strong inhibitory effect on pediatric rEPNs? Apatinib can significantly inhibit intracellular autocrine VEGF signaling, while bevacizumab showed no effect.^[23]^ Furthermore, an enzyme experiment showed that apatinib was an even more selective inhibitor of VEGFR-2 than sunitinib, with IC_50_ values of 1 nM and 5 nM, respectively. More importantly, apatinib can inhibit the transport function of ABCB1 and ABCG2 to reverse multidrug resistance, which enhances the effect of conventional antineoplastic drugs.^[24]^

## 4. Conclusion

According to the results of the patient in this case report, apatinib with TMZ exhibited outstanding efficacy for children with rEPNs who did not respond to multiple chemotherapy drugs. The inspiring result suggests that it may be necessary to launch a Phase II clinical trial of apatinib with TMZ to further evaluate its efficacy on pediatric rEPNs.
